# Assessment of the ability of open- and closed-loop cueing to improve turning and freezing in people with Parkinson’s disease

**DOI:** 10.1038/s41598-018-31156-4

**Published:** 2018-08-24

**Authors:** Martina Mancini, Katrijn Smulders, Graham Harker, Samuel Stuart, John G. Nutt

**Affiliations:** 1 Department of Neurology, Oregon Health & Science University, 3181 SW Sam Jackson Park Rd, OP-32, 97239 Portland, OR USA; 2 Sint Maartenskliniek, Research Department, PO Box 9011, 6500 GM Nijmegen, The Netherlands

## Abstract

Turning impairments are common in Parkinson’s disease (PD) and can elicit freezing of gait (FoG). Extensive examination of open-loop cueing interventions has demonstrated that they can ameliorate gait deficits in PD; less is known about efficacy to improve turning. Here, we investigate the immediate effectiveness of open- and closed-loop cueing in improving turning characteristics in people with PD. Twenty-five subjects with and 18 subjects without FoG participated in the study. Subjects turned in place for one minute under single- and dual-task for 3 randomized conditions: (i) Baseline; (ii) Turning to the beat of a metronome (open-loop); and (iii) Turning with phase-dependent tactile biofeedback (closed-loop). Objective measures of freezing, such as % time spent freezing and FoG-ratio, significantly improved when turning with both open-loop and closed-loop cueing compared to baseline. Dual-tasking did not worsen FoG in freezers, but significantly slowed down turns in both groups. Both cueing modalities significantly improved turning smoothness in both groups, but reduced turning velocity and number of turns compared to baseline. Both open and closed-loop cueing markedly improved turning in people with PD. These preliminary observations warrant further exploration of vibrotactile closed-loop cueing to improve mobility in everyday life.

## Introduction

Difficulties turning while walking are common among people with Parkinson’s disease (PD)^1,2^, negatively affect functional independence, and together with gait difficulties have recently been shown to be a major risk factor for falls, institutionalization and death in PD^3^. Turning requires the central nervous system to coordinate body re-orientation towards a new travel direction while continuing with the ongoing step cycle and maintaining medio-lateral stability^4,5^. Laboratory studies of turning in people with PD have reported reduced speed, increased turning duration, increased number of steps^1,6,7^, a narrower base of support^8^, and impaired segmental coordination of rotation (“en-bloc”)^4,5,9,10^. Turning has also been found as one of the strongest motor tasks to elicit Freezing of Gait (FoG) in the laboratory^11–14^. Over half of patients with PD develop FoG, an intermittent failure to initiate or maintain walking^15^. Although some motor changes in PD can be explained by decreased dopamine in the nigro-striatal pathway, dopaminergic treatments only partially improve gait disturbances and FoG, and sometimes produce a negative effect suggesting the involvement of other brain structures^16,17^. Therefore, non-pharmacological interventions, such as cueing, are used within clinical practice to help alleviate gait deficits and FoG.

Cues are temporal or spatial stimuli that regulate and facilitate repetitive movements by providing explicit motor targets^18,19^. Auditory, visual and tactile cues are often used to alleviate gait impairments in PD. Indeed, cueing such as clapping of the hands, a military march or visual stimuli placed on the floor often alleviates FoG^19^. Recently, Ginis *et al*., reviewed state-of-the-art cueing for subjects with PD who experience FoG^20^. Cues are predominantly used in an open-loop (constant rhythmical stimulus) rather than a closed-loop (intermittent stimulus set to individuals walking pattern) manner. Open-loop visual cues via Laser Canes^21^ and Google Glasses^22^ have an immediate effect on gait and health-related quality of life. Similarly, open-loop auditory cueing in PD with and without freezing improve turning characteristics, such as number of steps, duration and cadence as well as FoG^23,24^. However despite most studies finding positive effects of cueing for FoG in the short-term, differences have been found depending on disease profile when focusing on consolidation of learning and transfer toward untrained task^20^. There is also a lack of evidence that open-loop cueing has long-term effects on gait in PD.

More recently, novel closed-loop cueing devices have been developed by using wearable technologies to measure gait^25–28^. Closed-loop cueing, through a biofeedback paradigm, a way to provide information about a certain motor performance, that would otherwise be unknown to patients in real-time, has the potential to compliment internal feedback and reinforce weak or absent sensory signals^20,29^. In contrast to open-loop cueing, closed-loop feedback may lead to long-term motor skill learning and enhancement of adaptive cerebral plasticity^27,30,31^. Two recent studies have shown a residual effect of closed-loop biofeedback (visual or auditory) over open-loop biofeedback after 2 or 4 weeks of training on gait in subjects with PD^27,30^. However, tactile closed-loop cueing that provides additional feedback related to proprioception may be particularly effective for patients with FoG. Accumulating evidence suggests that inadequate integration of sensory inputs and defective proprioceptive internal maps may have a profound impact in FoG^32–34^. For instance, most patients with PD and FoG have no difficulties crawling or bicycling^35,36^, and when seated or lying down, they usually have mild or little difficulties in mimicking stepping movements. This context-specific FoG suggests that FoG occurs in specific conditions in which proprioception and postural adjustments are essential for the preparation and execution of locomotion^37,38^.

Our hypothesis is that enhancing proprioceptive stimuli, in the form of tactile biofeedback, may be effective in improving sensory integration and therefore alleviating turning impairments and FoG, in PD. Recently, De Nunzio^39^ demonstrated an increased stride length, cadence, and consequently increased velocity in PD patients exposed to trunk vibratory stimulation. Novak^37^ performed a pilot study assessing the short-term effects of step-synchronized vibration stimulation to the plantar region of the feet of a group of subjects with PD and a group of healthy elderly subjects. They showed improvements of walking speed, stride period, stride length, and cadence. However, to the best of our knowledge, no studies investigated the effectiveness of closed-loop biofeedback for turning. Here, we investigated the feasibility of enhancing proprioceptive stimuli, through closed-loop cueing, of improving turning and FoG in PD. We also investigated the effects of a well-known open-loop cueing (metronome) on improving turning and FoG.

## Results

The freezer and non-freezer groups showed similar age, cognitive status, disease duration and severity, as measured by the MDS-UPDRS Part III, MoCA, and feet sensation, see Table 1. However, the PIGD sub-score was significantly worse in the freezers.

**Table 1:** Subject characteristics in freezers and non-freezers group.

	Non-freezers N = 18	Freezers N = 25	p-value
Age (years)	70 ± 7	69 ± 7	0.9
Gender (% female)	22%	24%	0.5
Disease duration (years)	8.2 ± 4.7	9.3 ± 6.5	0.5
MOCA	26.2 ± 3.4	25.1 ± 4.6	0.4
MDS-UPDRS Part III	43.6 ± 11.6	47.1 ± 10.1	0.3
PIGD Subscore	3.8 ± 2.6	8.2 ± 3.8	0.001
Feet sensation (−8 to +8)	0.6 ± 6	2 ± 6	0.3

### Both open-loop and closed-loop cueing significantly reduced FoG while turning

**Figure 1:**
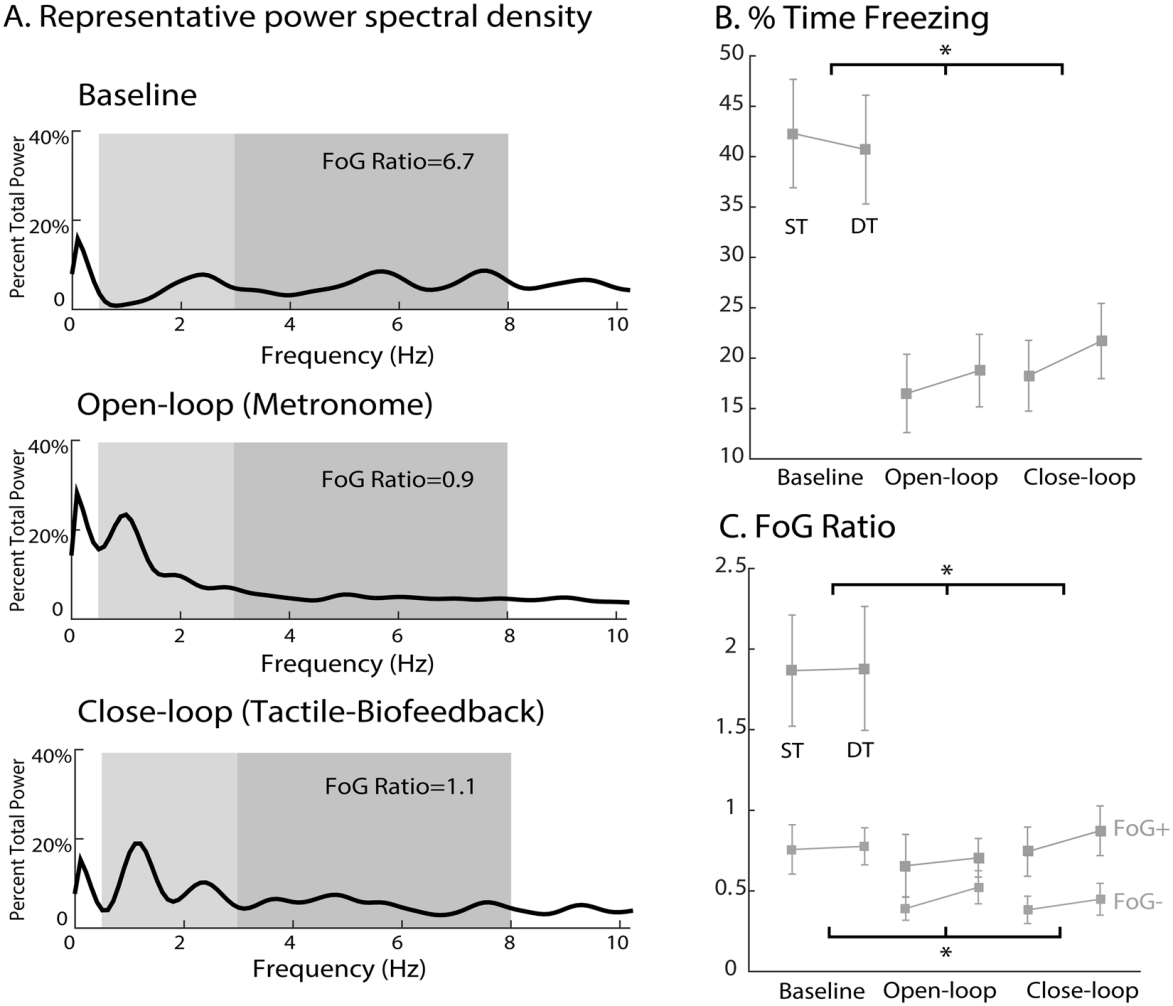
(**A**) Representative power spectral densities of the AP acceleration of the shins for baseline, open-loop, and closed-loop conditions together with the relative FoG ratio for that example. Right panel, freezing severity across the 3 conditions, measured by the (**B**) % time spent freezing (only in freezers) and (**C**) the FoG ratio (freezers and non freezers).

The % time spent freezing when turning significantly decreased with both cueing conditions under single and dual-task. For example; from 42 ± 26% (ST) and 33.9 ± 25% (DT) at baseline to 18 ± 20% (ST) and 18 ± 18% (DT) for open-loop cueing; and to 19 ± 18% (ST) and 18 ± 15% (DT) from the closed-loop cueing (conditions: F = 41, p < 0.0001; task: F = 0.4, p = 0.5; condition*task: F = 1.4, p = 0.2, Fig. 1).

Similarly, the freezing ratio significantly decreased in both the open-loop and closed-loop cueing conditions compared to baseline in both freezers and non-freezers (Fig. 1 and Table 2). In addition to a significant condition and group effect (F = 28, p < 0.0001; F = 7.4, p = 0.008), a significant condition*group interaction was found (F = 4.9, p = 0.03) indicating that freezers and non freezers benefit from cueing to a different degree.

**Table 2:** Turning characteristics (Mean and STD) in people with and without FoG in the 3 different conditions (baseline, open-loop, and close-loop).

	**Condition**	**Task**	Non freezers		Freezers							
			Mean	STD	Mean	STD						
FoG Ratio	Baseline	ST	0.78	0.66	1.86	1.69	**Condition**		**Group**		**Task**	
		DT	0.80	0.49	1.35	1.54	F-value	p-value	F-value	p-value	F-value	p-value
	Open-loop	ST	0.39	0.31	0.73	1.02	**28.244**	**<0.0001**	**7.417**	**0.008**	0.161	0.69
		DT	0.52	0.43	0.71	0.60	Condition*Group		Condition*Task			
	Closed-loop	ST	0.40	0.36	0.78	0.79	F-value	p-value	F-value	p-value		
		DT	0.47	0.42	0.69	0.43	**6.487**	**0.013**	1.017	0.317		
# of turns	Baseline	ST	13.65	4.51	7.88	3.98	**Condition**		**Group**		**Task**	
		DT	12.35	4.46	6.00	4.22	F-value	p-value	F-value	p-value	F-value	p-value
	Open-loop	ST	12.47	3.89	7.13	3.58	**28.838**	**<0.0001**	**40.962**	**<0.0001**	2.263	0.137
		DT	11.47	3.97	5.85	3.45	Condition*Group		Condition* Task			
	Closed-loop	ST	11.82	4.39	6.96	3.32	F-value	p-value	F-value	p-value		
		DT	10.76	4.05	5.70	3.44	**4.912**	**0.03**	0.977	0.326		
Average Peak Velocity (degrees/s)	Baseline	ST	141.76	38.65	100.29	29.13	**Condition**		**Group**		**Task**	
		DT	125.06	37.80	76.68	33.88	F-value	p-value	F-value	p-value	F-value	p-value
	Open-loop	ST	122.81	29.37	85.00	23.29	**52.838**	**<0.0001**	**35.187**	**<0.0001**	**4.975**	**0.029**
		DT	111.91	27.95	73.82	26.99	Condition*Group		Condition*Task			
	Closed-loop	ST	118.93	35.04	84.15	26.01	F-value	p-value	F-value	p-value		
		DT	105.03	29.81	72.99	26.51	**7.126**	**0.009**	3.128	0.081		
Average turn jerkiness (m^2^/s^5^)	Baseline	ST	0.31	0.16	0.58	0.44	**Condition**		**Group**		**Task**	
		DT	0.27	0.14	0.41	0.33	F-value	p-value	F-value	p-value	F-value	p-value
	Open-loop	ST	0.23	0.10	0.35	0.20	**16.476**	**<0.0001**	**10.302**	**0.002**	1.643	0.204
		DT	0.22	0.11	0.30	0.15	Condition*Group		Condition*Task			
	Closed-loop	ST	0.23	0.13	0.40	0.28	F-value	p-value	F-value	p-value		
		DT	0.21	0.11	0.32	0.18	1.526	0.221	1.015	0.317		

Linear mixed model results to investigate the condition, group, and task effects, as well the interaction condition*group and condition*task, are summarized on the right side of the table.

### Dual-tasking significantly reduced velocity of turns but not the total number of turns while cueing reduces both

Average turn peak velocity was lower in freezers compared to non-freezers (group effect: F = 35, p < 0.0001). Peak velocity significantly decreased in DT compared to ST (task effect: F = 4.9, p = 0.03) and with cueing (condition effect: F = 52, p < 0.0001). The condition*group interaction was significant (F = 7.1, p = 0.009), indicating again the different level of change in freezers and non freezers, while the group*task was not (F = 0.013, p = 0.9). The total number of turns was also lower in freezers compared to non-freezers (group effect: F = 40, p < 0.0001) and showed a decrease with cueing compared to baseline (condition effect: F = 28, p < 0.0001), but was similar under both ST and DT (task effect: F = 2.2, p = 0.13). In addition, the condition*group interaction was significant (F = 6.5, p = 0.01), while the group*task was not (F = 0.042, p = 0.8).

### Both open-loop and closed-loop cueing significantly reduced average jerkiness of turns

The average jerkiness during turning was significantly higher in freezers compared to non-freezers (group effect: F = 10, p = 0.002) and significantly decreased in both groups with cueing compared to baseline (condition effect: F = 16, p < 0.0001;). No task or interaction effects were significant, see Table 2.

### Dual-task cost was similar across the three turning conditions

The dual task cost was similar across baseline (6.5 ± 40%), open-loop (−3.2 ± 13.5%) and closed-loop (−2.3 ± 12.7%) cueing showing no significant condition (F = 2.7, p = 0.1), or group effect (F = 0.8, p = 0.3).

### Worse baseline performance was associated with greater benefit from cueing

**Figure 2:**
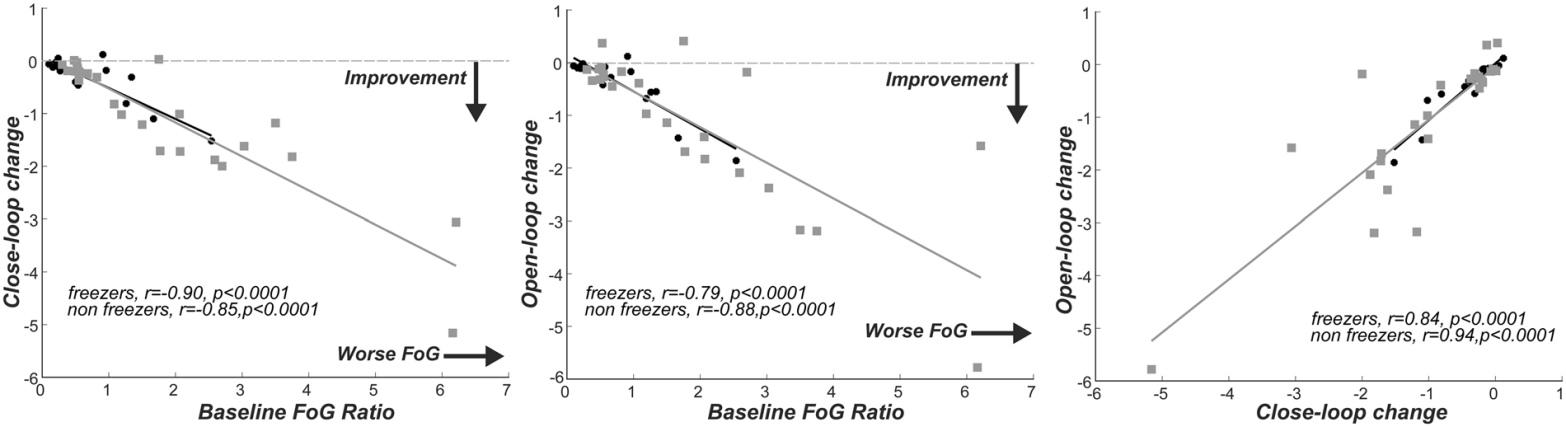
Association between the baseline FoG ratio at baseline and the delta change in the FoG ratio with the use of close- or open-loop cueing. In addition, association between the delta changes, close-loop cueing versus open-loop cueing has been reported in both freezers and non freezers.

In both freezers and non-freezers, worse FoG ratio at baseline related to higher improvement with both open-loop and closed-loop cueing (Fig. 2A,B). In addition, improvement with closed-loop cueing was associated with improvement with open-loop cueing in both groups (Fig. 2C).

### Patients’ perception (only freezers)

Results of subject impression on the efficacy of open-loop and closed-loop cueing on freezing while turning based on a Likert scale (7 points) showed that ~50% of the freezers reported an improvement in freezing using either the open-loop or closed-loop cues. Out of 25 subjects, 12 subjects expressed a clear preference for the closed-loop cueing, 6 for the open-loop cueing, and 7 didn’t have a preference. In addition, 4 subjects out of the 12 were interested in having the closed-loop biofeedback at home. Patient perception was not significantly associated to a change with open- or closed-loop cueing while turning.

## Discussion

This study showed a marked improvement in certain measures of turning quality, freezing and smoothness, and decreased speed of turning while using either open-loop (metronome) or closed-loop (tactile biofeedback) cueing in people with PD. In addition, neither the metronome nor tactile biofeedback seems to compromise the ability to carry out a concurrent cognitive task while turning. These findings were accompanied by the subjects’ positive perception on the efficacy of the cueing modality in reducing FoG on a Likert scale.

We demonstrated a marked reduction of FoG severity and percentage time spent freezing during turning on objective measures of FoG indicating that both open-loop and closed-loop cues were similarly effective in reducing freezing in the laboratory (immediate effect). This partially agrees with previous research that has shown that open-loop cues improve FoG measured using subjective patient or clinical rating within the laboratory^13,40,41^. However, as subjective scales are prone to error or bias, here, an objective, validated measure of FoG has been used to provide a more robust measurement of the effectiveness of cueing strategy.

In addition, we showed significant changes on other aspects of turning when using cueing in both freezers and non-freezers. Interestingly, cueing reduced the number of turns, and the average velocity of turning, but it significantly improved turning smoothness. Turning smoothness, measured by medio-lateral jerk while turning, has been previously found to be higher in freezers compared to non-freezers in both a similar study but different cohort of people^11^, and in another study measuring turning during community-living for 72 hours^42^. Reduced smoothness during turning and while freezing could reflect a higher number of steps to complete the turn and an increased amount of trunk corrections while turning in freezers^42^. Increased amount of trunk correction could be due to abnormal lateral anticipatory postural adjustments in freezers compared to non-freezers, consistent with previous work on turning and stepping in place^11,43^. Consistent with finding a reduction in freezing, smoothness of turning also improved with both open-loop and closed-loop cueing.

Reduction in number of turns and average velocity of turning with cueing compared to baseline may suggest that both freezers and non-freezers show a more cautious behavior while turning with cues. Previous studies investigating the role of auditory open-loop cueing for turning have also reported a marked decrease in FoG together with an increase in turning duration^23,24^. Although a reduced turning velocity has been previously shown in PD compared to healthy controls^11,44^, and levodopa replacement significantly increases turning velocity to control values^44^, it isn’t clear whether such increase is necessarily positive in people with PD. In fact, we recently showed impaired dynamic stability in people with PD compared to healthy controls only when walking and turning at fast speed^45^. Therefore, slower turns may represent a compensatory strategy to avoid instability^45^.

Although we did not directly compare the two cueing modalities, our findings showed that open- and closed-loop cueing were similarly effective in changing turning characteristics and alleviating FoG in people with PD. This result was not surprising, as there is a strong body of literature showing immediate, positive effects of open-loop cueing in PD^19,20^. However, while open-loop cueing may lose part of its effectiveness in the long-term^19,20^, few recent studies highlighted the potential learning effects with closed-loop cueing^27,30^. Here, by finding similar results in using closed-loop and open-loop cueing, we are more confident in proceeding with further studies investigating whether this closed-loop biofeedback may be of help in the long-term to improve turning characteristics in people with PD.

Global cognitive function, measured by the MoCA, was similar in freezers compared to non-freezers, and there was no significant association between global cognitive function and the change in freezing and quality of turning with either cue. This is in keeping with previous results showing that overall cognition wasn’t associated to the response of visual cueing on freezing^25^, but more in depth assessment of more specific executive-attentional test may be needed to rule out the dependency of the response to cueing to cognitive function.

A recent systematic review on cueing reported that gait characteristics, turning execution, dual-task performance, freezing and falls incidence were all improved in PD with cues in the laboratory^46^. However, the mechanisms underlying cue response are poorly understood, with theories suggesting that external cues may shift gait control from automatic to more voluntary conscious control (i.e. attention drawn to each step)^47^. If that is the case, cues may activate goal-directed or stimulus-driven fronto-striatal and fronto-parietal attentional pathways to by-pass sub-cortical deficits and overcome impairments^48^. Hence, further cortical activation would be required, particularly at the prefrontal cortex and posterior parietal cortex, to help with internal planning, updating and executing appropriate scaling and timing of gait characteristics (i.e. steps) for navigation through complex environments^49^. However, preliminary evidence of the activity of the prefrontal cortex while turning and freezing may partly be in contrast with this theory. Previous findings from Maidan *et al*.^50^ and from our laboratory showed that prefrontal activity is higher while turning and freezing, consistent with an increase in cognitive demands during freezing and the interference theory^51^. According to the interference theory^50,51^, it could be possible that the additional demands of turning (either cognitive or sensory overload) could lead to activation of the basal ganglia indirect route (controlled by the prefrontal cortex) over the direct route (controlled by the striatum) resulting in freezing. Therefore, according to this model, cueing might improve motor automaticity by reducing the demand on the indirect route, improving sensory integration, and favoring the activation of the direct route reducing FoG and other gait disturbances.

Impaired motor automaticity in PD has been generally overlooked and less investigated in comparison to other motor deficits^52^. People with PD appear to lose previously stored automatic skills due to the impairment of the sensorimotor striatum likely causing an increase demand on the prefrontal cortex in order to execute basic motor operations via attentional processes^52,53^. Therefore, FoG and turning improvements with cueing might result from decreased cortical control of turning due to augmentation of peripheral drive.

Limitations of the current study were testing only OFF dopaminergic medication and a different modality for the controlled cueing condition (audio metronome versus a tactile-like metronome). Our preliminary observations suggest that augmenting somatosensory information with a phase-dependent biofeedback system relying on an unobtrusive modality, might be an effective tool in reducing FoG and improving turning quality. Future studies will investigate if these positive results will translate for long-term use in community-living environment.

## Methods

### Participants

Forty-three subjects with PD were recruited through the Parkinson’s Center of Oregon clinic at Oregon Health & Science University.

Inclusion criteria were: Diagnosis of idiopathic Parkinson’s disease with sensitivity to levodopa and off-medication Hoehn & Yahr scores of II-IV. Exclusion criteria: Other factors affecting gait (hip replacement, musculoskeletal disorder, uncorrected vision or vestibular problem), or an inability to stand or walk for 2 minutes at a time. Individuals were excluded if they could not safely walk 20 feet without walking aids, or if they had any musculoskeletal or vestibular disorder, and dementia.

Of the 43 PD patients, 25 were classified as freezers based on a score of >0 on the New Freezing of Gait Questionnaire (NFOGQ)^54^. Eighteen subjects scoring 0 were classified as non-freezers. All PD patients were tested in the “OFF” state, after at least 12-hour overnight withdrawal from anti-parkinsonian medications.

This study was carried out in accordance with the recommendations of the Oregon Health & Science University (OHSU) institutional review board (IRB) with written informed consent from all subjects. All subjects gave written informed consent in accordance with the Declaration of Helsinki. The protocol was approved by the OHSU IRB (#9903).

### Protocol

After explaining the study and obtaining informed consent, the participants underwent a 3-hour assessment, which included clinical assessments, questionnaires, and quantitative assessments of balance and gait.

The participants repeated three blocks of the same motor task under three different conditions: (1) no cues (baseline condition), (2) closed-loop cueing (phase-dependent foot vibration biofeedback), (3) open-loop cueing (fixed auditory metronome, pace chosen by the subject). Each condition included a two-minute walk in a 8 m hallway, a one-minute turning in place, both repeated with and without a concurrent cognitive task, and a figure 8 task through a doorway. Condition order was randomized across subjects (e.g. 1, 2, 3 or 1, 3, 2) using a blocked randomization strategy so that approximately equal numbers of subjects received each ordering. Between conditions the subjects were asked to take a break of 10–15 minutes in order to avoid adaptation from the previous condition. In addition, at each break the participants were asked to rate in a Likert scale the efficacy of the open-loop and closed-loop cueing on mobility and freezing.

While performing the abovementioned motor tasks, participants wore 8 wireless, synchronized inertial sensors (Opals by APDM, Inc) on both shins, feet, wrists, on the sternum and on the posterior trunk (around L5). For the turning in place test, subjects stood and turned in place alternating 360° turns to their right, then 360° to their left, repeating this sequence at their fastest speed. The same test was repeated while subtracting 3′s from a 3digit number. The inertial sensors recorded 3-D linear accelerations and 3-D angular velocities at 128 Hz. Data were stored for offline analysis with Matlab.

Closed-loop condition: VibroGait, a wearable system, previously described^28^ was used to deliver tactile stimuli to the wrist while in the stance phase of gait. Briefly, the system^28^ plugs into the Opal placed on the shins and consists of a novel controller unit (Arduino microcontroller) that senses through a gyroscope when the foot is on the ground and activate the tactor unit to generate a vibration (in our case to the wrist). The tactors are C-2 tactors (Engineering Acustic, Inc) with a primary resonance in the 200–300 Hz range. The vibration intensity is similar to that of a cell phone operating in vibration mode.

Open-loop condition: an auditory tone^19,40^ was delivered through a portable speaker placed in the center of the laboratory so that it was possible to clearly hear the tone while performing the motor tasks. Subjects were asked to synchronized each step with the auditory cue and they self-selected the pace of the cue.

### Clinical, cognitive and sensory assessment

Clinical assessment involved the Motor Section (III) of the Unified Parkinson’s Disease Rating Scale (MDS-UPDRS), which consists of 23 items related to bradykinesia, rigidity, tremor, and posture and gait signs rated on a 4-point scale^55^, at the end of the mobility assessment. The Posture Instability and Gait Disability subscore (PIGD) was also calculated from the MDS-UPDRS Part III^56^.

Global cognition was examined using the Montreal Cognitive Assessment (MoCA)^57^.

Vibratory sensation was tested with a 128-Hz tuning fork at the interphalangeal joint of the hallux, 5^th^ metatarsal head, arch/plantar surface and ankle for both the right and left foot^58^. The examiner stroked the end of the tuning fork hard enough that the sides touched, and immediately placed the vibrating tuning fork firmly on the bony prominence of the site of interest. The patient was instructed to tell the examiner when the patient felt the vibration start and stop. The examiner waited about 5 s for the patient to perceive the vibration. The patients’ perception was scored as absent, reduced, or normal. Note that before the examinations, the examiner applied the vibrating tuning fork on the patient’s wrist, to make sure that the patient could recognize the vibration. The sensation was summarized with a composite score were −8 is absent sensation and +8 normal sensation in all the 4 right and left foot locations.

### Objective measures

**Figure 3:**
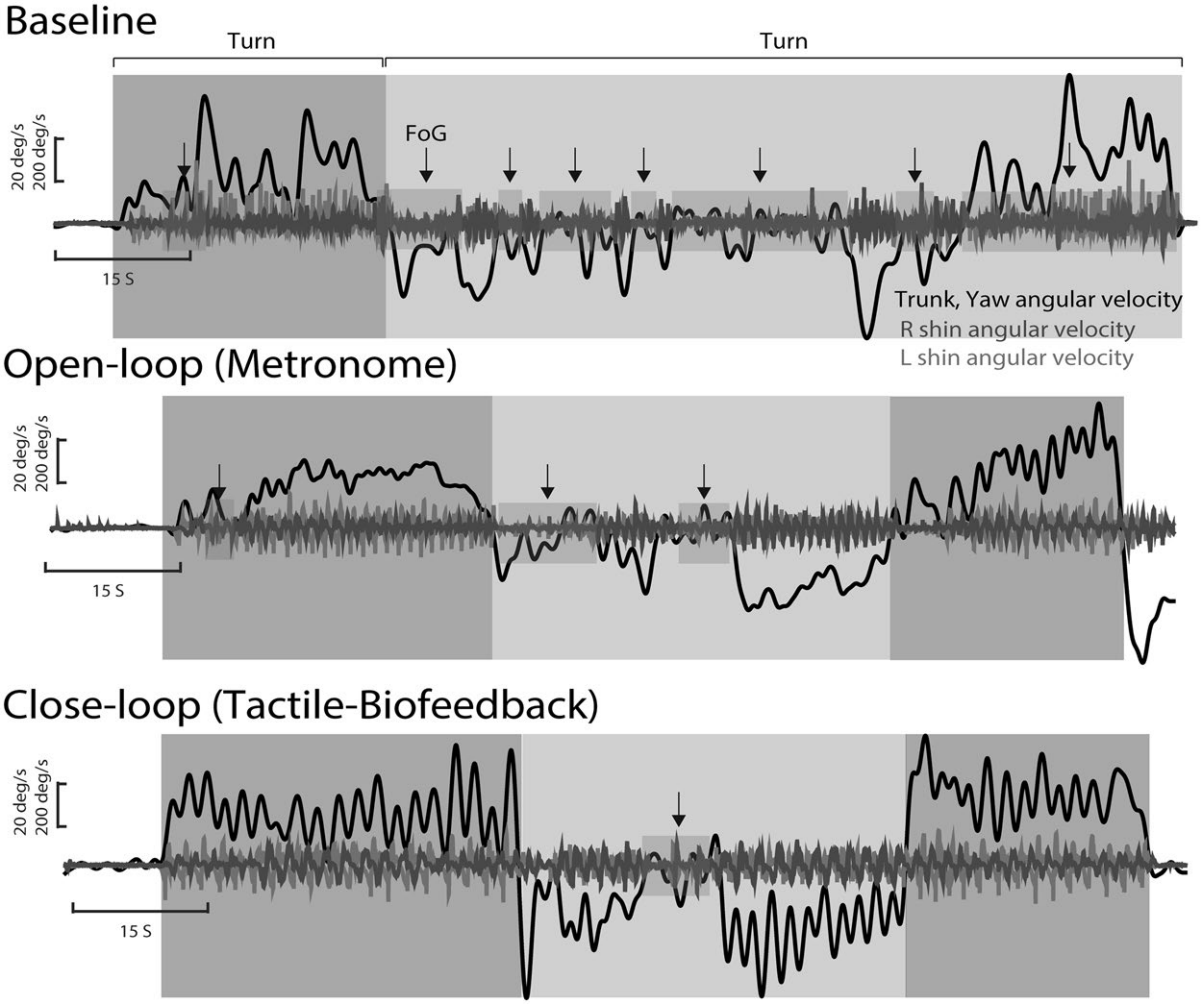
Representative data in a subject with FoG from trunk and shins sensors while turning during baseline, open-loop cueing, and closed-loop cueing. Grey shaded areas show turn duration while pink areas show data during freezing of gait episodes.

The following measures were extracted to objectively characterize freezing and turning for each conditions and were previously described and validated in Mancini *et al*. 2017^11^: (1) FoG ratio as index of freezing severity calculated as the power spectral density ratio between high (3–8 Hz) and low (0–3 Hz) frequencies of antero-posterior shin accelerations, (2) the percentage of time spent freezing during the task, calculated as the time in which the FoG ratio was higher than 1 (for either right or left foot), (3) number of turns from yaw angular velocity of the sensor on the posterior trunk, (4) the average turn peak velocity, from the yaw angular velocity of the sensor on the posterior trunk, and (5) the average jerkiness of the turns, quantifying fluidity of turning. Examples of representative signals during the task are represented in Fig. 3.

### Statistical Analysis

Independent sample t-tests compared age, disease duration, MDS-UPDRS III, PIGD, and MoCA between the two groups. To investigate whether freezing and turning were changing with the use of open-loop and closed-loop cueing, linear mixed models were fit for our outcome measures and main effects of condition (baseline, open-loop, closed-loop), group (freezers and non-freezers), task (single-task, ST; dual-task, DT) were reported. The condition*group interaction and the group*task interaction terms were included to test whether groups had different linear differences between condition and task. Each model included a random intercept for each subject to account for the repeated measurements within each subject. To evaluate the performance on the cognitive task, the number of correct answers was derived for the seated condition, as well as for the baseline, open-loop and closed-loop turning conditions. The dual-task cost for the three turning conditions was then calculated as the percent change relative to the seated performance (%change = (turning-seated)/seated*100). A similar model was fit to test if the dual-task cost differed among baseline, open-loop and closed-loop turning between freezers and non-freezers.

We used linear regressions to assess whether the change in freezing and turning characteristic with open-loop or closed-loop (from baseline) was associated with disease duration, severity, or cognitive function in PD. All statistical analysis was performed in MATLAB r2016b (The Mathworks Inc., Natick MA, USA).

The datasets generated during and/or analysed during the current study are available from the corresponding author on reasonable request.
